# Diagnosis, Prognosis and Treatment of Canine Cutaneous and Subcutaneous Mast Cell Tumors

**DOI:** 10.3390/cells11040618

**Published:** 2022-02-10

**Authors:** Andrigo Barboza de Nardi, Rodrigo dos Santos Horta, Carlos Eduardo Fonseca-Alves, Felipe Noleto de Paiva, Laís Calazans Menescal Linhares, Bruna Fernanda Firmo, Felipe Augusto Ruiz Sueiro, Krishna Duro de Oliveira, Silvia Vanessa Lourenço, Ricardo De Francisco Strefezzi, Carlos Henrique Maciel Brunner, Marcelo Monte Mor Rangel, Paulo Cesar Jark, Jorge Luiz Costa Castro, Rodrigo Ubukata, Karen Batschinski, Renata Afonso Sobral, Natália Oyafuso da Cruz, Adriana Tomoko Nishiya, Simone Crestoni Fernandes, Simone Carvalho dos Santos Cunha, Daniel Guimarães Gerardi, Guilherme Sellera Godoy Challoub, Luiz Roberto Biondi, Renee Laufer-Amorim, Paulo Ricardo de Oliveira Paes, Gleidice Eunice Lavalle, Rafael Ricardo Huppes, Fabrizio Grandi, Carmen Helena de Carvalho Vasconcellos, Denner Santos dos Anjos, Ângela Cristina Malheiros Luzo, Julia Maria Matera, Miluse Vozdova, Maria Lucia Zaidan Dagli

**Affiliations:** 1 Department of Veterinary Clinic and Surgery, Universidade Estadual Paulista (UNESP), Jaboticabal 14884-900, Brazil; andrigobarboza@yahoo.com.br (A.B.d.N.); n-paiva@hotmail.com (F.N.d.P.); laiscmlinhares@gmail.com (L.C.M.L.); denner.anjosoncology@gmail.com (D.S.d.A.); 2 Department of Veterinary Medicine and Surgery, Veterinary School, Universidade Federal de Minas Gerais, Belo Horizonte 31270-901, Brazil; rodrigohorta@ufmg.br (R.d.S.H.); paulopaes@vet.ufmg.br (P.R.d.O.P.); 3 Institute of Health Sciences, Universidade Paulista (UNIP), Bauru 17048-290, Brazil; carlos.e.alves@unesp.br; 4 Department of Veterinary Surgery and Animal Reproduction, Universidade Estadual Paulista (UNESP), Botucatu 18618-681, Brazil; 5 Department of Veterinary Medicine, Universidade Federal do Paraná, Curitiba 80035-050, Brazil; brunaf_vetxlv@hotmail.com; 6 Histopathological Diagnosis Department, VETPAT—Animal Pathology & Molecular Biology, Campinas 13073-022, Brazil; felipesueiro@hotmail.com; 7 Surgical Pathology, Vet Câncer LTDA-ME, São Paulo 04523-013, Brazil; vclabpathology@gmail.com; 8 General Pathology Department, Dental School, Universidade de São Paulo (USP), São Paulo 05508-000, Brazil; silvialourenco@usp.br; 9 Laboratory of Comparative and Translational Oncology (LOCT), Department of Veterinary Medicine, Faculty of Animal Science and Food Engineering, Universidade de São Paulo (USP), Pirassununga 13635-900, Brazil; rstrefezzi@usp.br; 10 Institute of Health Sciences, Universidade Paulista (UNIP), São Paulo 01533-000, Brazil; carlosbrunner@hotmail.com; 11 Clinical and Surgical Oncology, Vet Cancer Animal Oncology and Pathology, São Paulo 04523-013, Brazil; diretoriavetcancer@gmail.com; 12 Clinical Oncology, Onccarevet e Onconnectionvet, Ribeirão Preto 14026-587, Brazil; paulocjark@hotmail.com; 13 Técnica Cirúrgica da Pontifícia, Pontifícia Universidade Católica do Paraná (PUCPR), Curitiba 80215-901, Brazil; castrojlc@jorgecastrovet.com; 14 Clinical and Surgical Oncology, E+ Veterinary Specialties, São Paulo 04078-012, Brazil; ubukata@gmail.com (R.U.); karen.batschinski@gmail.com (K.B.); 15 Clinical, Surgical and Palliative Care Oncology, Onco Cane Veterinary, São Paulo 04084-002, Brazil; renatasobral@oncocane.com; 16 Radiation Oncology Service, Pet Care Oncologic Center, São Paulo 05659-010, Brazil; nataliacruz.vet@gmail.com; 17 Naya Specialties, Campo Belo 04608-003, Brazil; adriananishiya@hotmail.com; 18 SEOVET—Specialized Service in Veterinary Oncology, Clinical and Surgical Oncology, São Paulo 05016-000, Brazil; sicrestoni@gmail.com; 19 Clinical Oncology and Radiation Therapy, Oncopet Veterinary, Rio de Janeiro 22631-000, Brazil; Simonecsc@gmail.com; 20 Department of Animal Medicine, Veterinary School, Universidade Federal do Rio Grande do Sul, Porto Alegre 91540-000, Brazil; daniel.gerardi@ufrgs.br; 21 ONE Patologia Veterinária, Santos 11055-051, Brazil; onepatovet@gmail.com; 22 Small Animal Internal Medicine Department, School of Veterinary Medicine, Universidade Metropolitana de Santos (UNIMES), Santos 11045-002, Brazil; lrbiondi@gmail.com; 23 Department of Veterinary Clinic, School of Veterinary Science and Animal Health, Universidade Estadual Paulista (UNESP), Botucatu 18618-681, Brazil; Renee.laufer-amorim@unesp.br; 24 School of Veterinary, Universidade Federal de Minas Gerais, Belo Horizonte 31270-901, Brazil; gleidicel@yahoo.com.br; 25 Surgery Department, Univet Veterinary Clinic—São José Do Rio Preto, São José do Rio Preto 15085-420, Brazil; rafaelhuppes@hotmail.com; 26 Vetschool São Paulo: Veterinária, Universidade Estadual Paulista (UNESP), São Paulo 03308-010, Brazil; fgrandivet@gmail.com; 27 Surgical and Clinical Oncology, Bota Fogo Veterinary Hospital, Rio de Janeiro 22281-180, Brazil; carmenvasconcellos@hotmail.com; 28 Eletro-Onkovet Service, Franca 14406-005, Brazil; 29 Department of Surgery, Medical Sciences College, Universidade Estadual de Campinas (UNICAMP), Campinas 13083-970, Brazil; angela.luzo@gmail.com; 30 Department of Surgery, School of Veterinary Medicine and Animal Science, Universidade de São Paulo (USP), São Paulo 05508-270, Brazil; materajm@usp.br; 31 Veterinary Research Institute (VRI), 621 00 Brno, Czech Republic; vozdova@vri.cz; 32 Department of Pathology, School of Veterinary Medicine and Animal Science, Universidade de São Paulo (USP), São Paulo 05508-900, Brazil

**Keywords:** cutaneous, dog, mast cell tumor, subcutaneous

## Abstract

Mast cell tumors (MCTs) are hematopoietic neoplasms composed of mast cells. It is highly common in dogs and is extremely important in the veterinary oncology field. It represents the third most common tumor subtype, and is the most common malignant skin tumor in dogs, corresponding to 11% of skin cancer cases. The objective of this critical review was to present the report of the 2nd Consensus meeting on the Diagnosis, Prognosis, and Treatment of Canine Cutaneous and Subcutaneous Mast Cell Tumors, which was organized by the Brazilian Association of Veterinary Oncology (ABROVET) in August 2021. The most recent information on cutaneous and subcutaneous mast cell tumors in dogs is presented and discussed.

## 1. Introduction

Several epidemiological studies from many countries point out that mast cell tumors (MCTs) have a high frequency in dogs, and is thus important in veterinary oncology. In dogs, it is the third most common tumor subtype, and is the most common malignant skin tumor, accounting for 11% of skin cancer cases [1,2,3,4,5,6,7,8,9,10,11,12,13,14]. Access to up-to-date information about diagnostic techniques, treatment, and prognosis is extremely important to ensure an optimized approach and improve the outcomes for the patient. 

The 1st Brazilian consensus meeting on canine MCTs occurred in 2012, and the results were reported by Nardi et al. in 2018 [14]. The present review is the result of the 2nd Consensus Meeting on the Diagnosis, Prognosis, and Treatment of Canine Cutaneous and Subcutaneous Mast Cell Tumors organized by the Brazilian Association of Veterinary Oncology (ABROVET) in August 2021. The present review presents the most recent information on cutaneous and subcutaneous mast cell tumors in dogs. The work was prepared by veterinarians working in the field of veterinary oncology from across Brazil and from the Czech Republic (Miluse Vozdova). This review aims to present a complete report to all scientists interested in mast cell diseases. 

### 1.1. Incidence and Etiology

MCT is recognized as one of the most common skin tumors in dogs [15]. Studies perforMed. in Brazil have described an incidence ranging between 20.9% and 22.4%, revealing MCT as the second-most frequent malignant neoplasm in dogs, after mammary gland tumors [16,17,18].

Some breeds are predisposed to MCT development, including Boxer; Bull Terrier; French Bulldog; Golden Retriever; Labrador Retriever; Shar-pei; and Dachshund [16,19,20,21,22,23,24,25,26,27]. On the other hand, some breeds present a lower risk of MCT development, including the German Shepherd, Chihuahua, Poodle, Yorkshire Terrier, and Cocker Spaniel [24,25]. Recent studies also sought to correlate the breed predisposition to the biological behavior of MCT, and suggested that Pug and Boxer dogs are more prone to tumors with less aggressive behavior, while the shar-pei tends to develop more aggressive tumors [15,24,25].

In terms of sexual predisposition, results have been contradictory. To date, no sexual predisposition has been considered. In terms of age groups, MCT can develop at any age, but it is more common in adult to older animals [15,25].

The etiology of MCT has not been completely elucidated. However, Vail et al. [15] suggested the influence of chronic inflammation in the skin and exposure to irritating compound. Furthermore, the presence of mutations in the c-KIT gene (*KIT*) has been related to tumor development in MCT cases. This gene encodes a receptor tyrosine kinase that binds stem cell factor (SCF) in canine mast cells. Mutations drive uncontrolled cell survival and proliferation, which is related to MCT development and progression [28,29,30,31,32].

### 1.2. Genetics of Canine MCTs

Although mast cell tumors are among the most common cancers in dogs, the genetic basis of their origin and development has not been fully elucidated. Mutations in *KIT*, which encodes the c-KIT tyrosine kinase receptor, have been found in 10–45% of cutaneous MCTs in dogs. The most common type of mutation is an internal tandem duplication (ITD), typically in exons 8 or 11 of *KIT*, followed by point mutations in exons 8, 9, 11, and 17 [28,30,33,34,35,36,37,38]. The KIT transmembrane receptor is involved in cell differentiation and proliferation. Most known KIT mutations cause constitutive activation of the KIT receptor in the absence of its ligand, i.e., SCF. Mutations in *KIT*, other than those in exon 8, are associated with aggressive behavior of MCTs, reduced survival time, and increased incidence of tumor-related recurrence and death [11,30,32,39,40,41,42].

In addition, mutations in the *GNB1* (17.3% of MCTs), *TP53* (14.6% of MCTs), and several other genes have been identified at low frequency in canine cutaneous MCTs [41,43,44]. *TP53* is a known tumor suppressor gene mutated in a number of human and animal tumors, although *TP53* mutations have not proven to be reliable prognostic markers in canine MCTs [41]. *GNB1* encodes a subunit of the guanine nucleotide-binding protein. Mutations in *GNB1* have been found to be associated with MAPK and anti-apoptotic pathways in human leukemia [45]. In dogs, *GNB1* mutations have been detected in cutaneous and subcutaneous MCTs, with a trend towards a positive prognosis [41].

Regarding cytogenetic abnormalities, copy number variations in several chromosomes and chromosomal translocations have been detected in canine MCTs by Array CGH and fluorescence in situ hybridization. Specifically, loss of chromosome 5, gain of chromosome 31, and partial gain or loss of chromosome 20 were found to be associated with aggressive MCTs [46]. Interestingly, copy number loss of *PTEN* and *FAS*, as well copy number gains of *MAPK3*, *WNT5B*, *FGF*, *FOXM1*, and *RAD51* have previously been associated with poor outcomes in MCT-affected dogs [47]. 

In summary, *KIT* mutations (excluding mutations in exon 8), multiple aneuploidy, polyploidy, and accumulation of gene mutations and chromosomal and subchromosomal copy number variants are associated with aggressive, higher-grade MCTs and a negative prognosis. However, further research is needed to elucidate the etiology of MCTs and to discover new reliable prognostic markers and targets for therapy.

### 1.3. Biological Behavior

**Figure 1:**
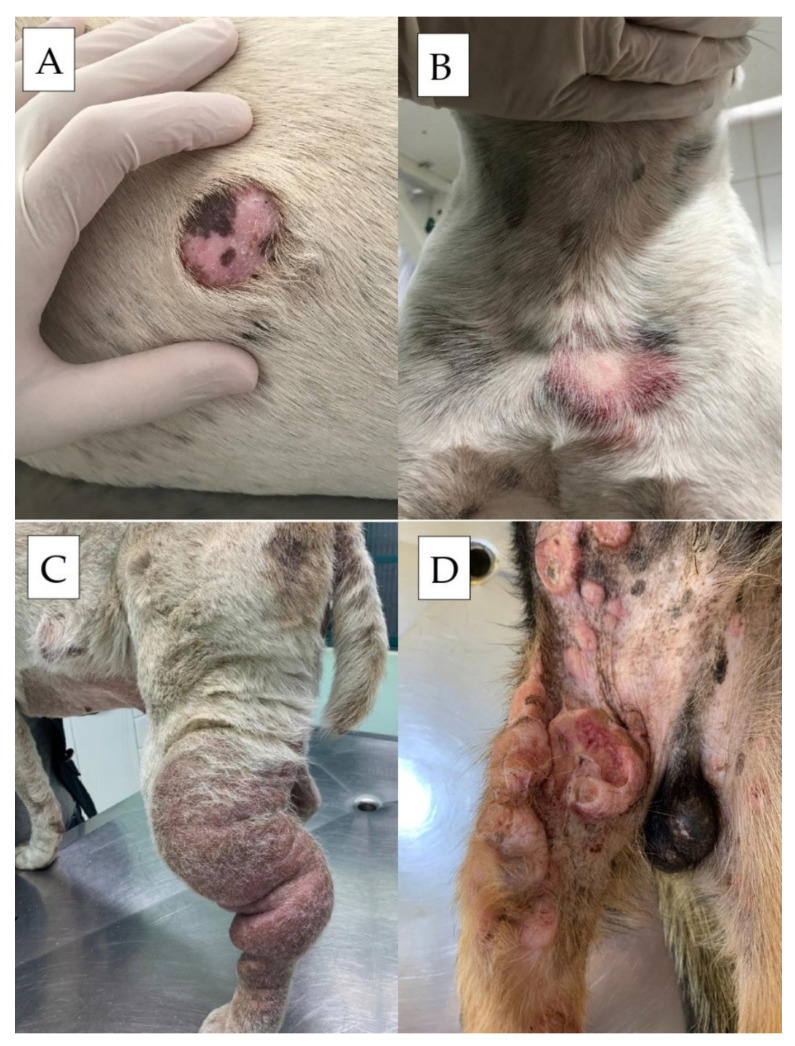
Canine mast cell tumors evidencing different clinical presentations. (**A**) Solitary, well-delimited and alopecic lesion in a dog. (**B**) Solitary irregular erythematous and partially alopecic cutaneous lesion. (**C**) Disseminated lesion with cutaneous/subcutaneous involvement and limb edema. (**D**) Multiple lesions with ulceration aspect.

The biological behavior of MCTs in dogs is considered to be extremely variable. In general, according to Patnaik et al. [48], when MCTs are well-differentiated, they present a milder behavior. In contrast, less-differentiated tumors have a more aggressive behavior. The organs most frequently affected by metastases are the lymph nodes, skin, spleen, and liver, and less frequently the lungs [15]. The metastatic potential of MCTs varies according to its histopathological classification, occurring in less than 10% of cases of well-differentiated mast cell tumors, in 5% to 22% of moderately differentiated neoplasms, and in 55% to 95% of poorly differentiated cases. Metastases occur mainly on locoregional lymph nodes, and may later affect the spleen, liver, and other organs. Patients with mast cell tumors of any degree and who have regional lymph node involvement in general have a poorer prognosis [20,21,49,50,51]. Clinically, canine MCTs can manifest as formations of different sizes and aspects, with widely variable presentation (Figure 1). They can be delimited (Figure 1A), elevated, firm, soft, pruritic, and can also present erythematous areas (Figure 1B), with invasion of the subcutaneous tissue (Figure 1C), and in up to 30% of cases, ulceration (Figure 1D). Some cases may present with irregular, elevated formations, with a soft consistency, without erythema and ulceration, and may macroscopically appear to be cutaneous lipomas. Cutaneous and subcutaneous manifestations are macroscopically similar and can only be differentiated by histopathological examination. In terms of location, about 50% of canine MCTs develop in the trunk, perineum, and inguino-genital regions, 40% occur in the limbs, and 10% in the head and neck [15,52].

MCTs most commonly develop as solitary lesions; however, multiple formations can occur, independently, or with disease progression, making it difficult to differentiate between these situations. Lesions can be considered separately, and prognostic factors should be assessed in relation to each tumor in isolation [20]. However, although O’Connell and Thompson [53] suggested that the presence of multiple MCTs does not influence overall survival, Kiupel et al. [19] have described that these cases have worse prognosis. Some cases may prove even more challenging because they have low histopathological grades, but are nevertheless associated with aggressive behavior, with regional or distant metastases already present at the time of diagnosis [54]. In contrast, some high-grade MCTs have less aggressive behavior and a favorable prognosis [55]. Subcutaneous MCTs tend to exhibit less aggressive behavior [2], although some veterinarians have reported contradictory observations. Although a recent publication describes the characteristics of cutaneous and subcutaneous tumors [56], the biological behavior of cutaneous and subcutaneous MCT have not yet been directly compared in a study. 

In terms of assessing location-related behavior, previous studies have reported that MCTs located in the oral cavity, nail bed, and inguinal, perineal, and preputial regions exhibited more aggressive behavior [57,58,59,60]. However, different studies have ruled out the association of this behavior with locations in the oral cavity and in the inguinal and perineal region [61,62]. MCTs located in the scrotum and nasal plane are more prone to higher grades and earlier metastasis [11,25]. In addition to the cutaneous and subcutaneous MCT forms, a visceral form is also considered, characterized by infiltration into the abdominal lymph nodes, liver, and spleen [15], and disseminated mastocytosis, when there is systemic involvement in the bone marrow [52]. However, these are considered uncommon presentations in dogs. 

Other forms of dissemination related to MCTs are mastocythemia (when mast cells are present in the peripheral blood), and mast cell leukemia (when there is exacerbated and uncontrolled proliferation of mast cells in the bone marrow). Both are considered uncommon and are mainly related to cases of advanced MCTs, although they can also occur in association with other illnesses [15,63,64,65].

### 1.4. Clinical Signs and Paraneoplastic Syndromes

In addition to the presence of tumor formations, secondary clinical signs of MCT are present in about half of affected dogs, and include signs resulting from mast cell degranulation, and consequent release of histamine, heparin, eosinophil chemotactic factor, and proteolytic enzymes [15,52,66]. The effects of degranulation can be observed during physical examination, when, after mechanical palpation, erythema, edema, and papule formation are observed in the region, in a manifestation called Darier’s sign. Delayed wound healing, coagulation abnormalities, and, less frequently, hypotension and circulatory collapse may also occur [15,52,66,67].

Gastrointestinal complications are also seen, with signs of ulceration mainly affecting the stomach and less frequently the duodenum. These lesions are usually multiple and superficial, although, in some situations, more severe ulcerations can occur. Its manifestation is attributed to high blood levels of histamine that stimulate the H2 receptor on parietal cells, resulting in excessive production of gastric acid and increased gastric motility. In addition, histamine damages the vascular endothelium of arterioles and venules and releases fibrolysin, leading to intravascular thrombosis and ischemic necrosis of the stomach mucosa. Heparin, in turn, tends to block the effects of histamine, but is present in low concentrations [52,66,67]. In these cases, clinical signs of hematemesis, anorexia, hematochezia, melena, anemia, abdominal pain, and, in severe cases, intestinal perforation, peritonitis, and sepsis can be observed [15,52,66,67]. According to Welle et al. [66], gastrointestinal ulcerations are observed in 35–83% of canines affected by MCTs, based on necropsy examinations.

The delay in healing and dehiscence, often observed after surgical resection of MCTs, are related to the release of vasoactive amines and proteolytic enzymes by mast cells, which, once linked to H1 and H2 receptors, can lead to the suppression of fibroblast growth factor, reducing the fibroplasia [52,66]. In addition, local bleeding can also be seen during surgical resection, probably due to coagulation deficits caused by heparin release [66]. Circulatory collapse cases, although rare, can occur in the presence of massive histamine release from neoplastic cells, particularly in animals with extensive disease [66,67]. The presence of hypereosinophilia is described as a paraneoplastic syndrome associated with MCTs in cases of cutaneous and visceral disease. Its manifestation is associated with the release of eosinophil chemotactic factors [68,69,70].

## 2. Diagnostic Approach

The diagnostic evaluation of dogs suspected of having MCTs aims to reach a definitive diagnosis through cytopathological, histopathological, and immunohistochemical examination; define complete clinical staging; identify concomitant paraneoplastic syndromes; and assess associated prognostic factors.

### 2.1. Cytological Analysis

**Figure 2:**
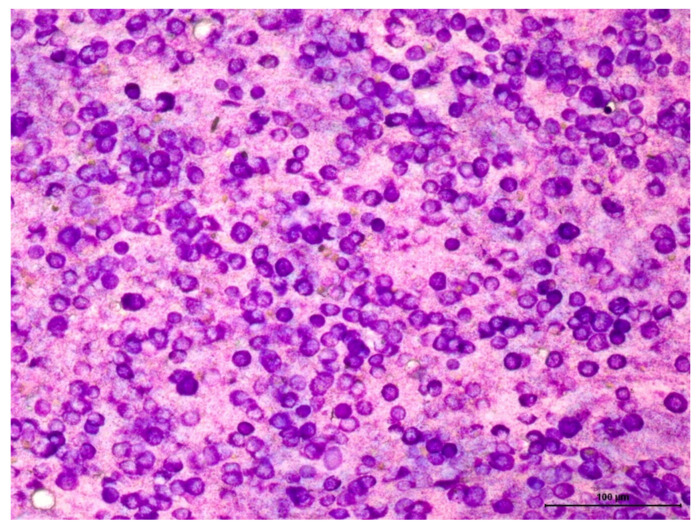
Cytological analysis of a cutaneous well-differentiated mast cell tumor stained with Giemsa. It is observed a round ell population, showing a high degree of cytoplasmic granulation. Granules are also observed in the slide background. Objective: 40×.

Fine-needle cytology is the basis of MCT diagnostic investigation and is often perforMed. during the initial consultation [1,53], achieving the correct diagnosis in 92–96% of cases (Figure 2) [67]. Tissue collection is usually perforMed. with a fine needle, with or without aspiration. The use of a 13 × 4.5 mm (26 G) needle is recommended in order to reduce blood contamination, thus increasing the accuracy of the diagnosis [71]. The technique is considered quick, less costly, and non-invasive, and is very useful for obtaining an early diagnosis [72], which facilitates treatment. In routine Brazilian veterinary pathology, there are two staining kits that are known to be useful, including the May–Grünwald–Giemsa and Instant Prov stains. Both are based on the Romanowski technique and are effective for staining mast cell granules. It should be noted that the Diff-Quik dye, frequently used in the USA and other countries, may not stain or only partially stain mast cell granules, making it difficult to diagnose MCTs [72,73,74,75]. In inconclusive cases, in which mast cell staining does not occur, the use of stains, such as Giemsa and toluidine blue, is recommended [72,74].

Among round cell neoplasms, MCTs have a characteristic cytomorphology, with the presence of fine to coarse basophilic intracytoplasmic granules. However, high-grade tumors may not present abundant cytoplasmic granulation. A possible explanation would be the loss of cell differentiation during the process of malignant transformation. Nevertheless, other potential causes for a reduction in the number of granules, such as spontaneous or palpation-induced degranulation, should also be considered. Furthermore, poorly differentiated tumors are more prone to degranulation and not necessarily to less granule production. Detailed studies on this subject are lacking. Strefezzi et al. [73] demonstrated a correlation between the mean nuclear area and survival time in samples submitted to morphometric study.

Assessment of the tumor grade in cytological smears does not allow us to follow the grading system proposed by Patnaik et al. [48] strictly, which, in addition to cytomorphological criteria, also considers the level of dermal invasion not determinable by cytological examination. Among the various articles published, the study by Camus et al. [76] has been the most objective and was based on specific morphological criteria selected after statistical analysis. The selected algorithm that presented the highest correlation with Kiupel’s histological grade classified MCTs into two groups: high- and low-grade. The selected criteria were: at least one smear with adequate cellularity; granulation; presence or absence of mitotic figures; nuclear pleomorphism; presence or absence of binucleation or multinucleation; and >50% anisokaryosis.

Hergt et al. [77] also proposed criteria for a cytological classification system for MCTs, dividing tumors into low- and high-grade, and concluded that the classification is useful for the initial assessment, although the reliability of cytology is considered inadequate at this point. The proposed classification resulted in a sensitivity of 86.8% and a specificity of 97.1% for evaluating the agreement between histological and cytological grading. Nevertheless, five high-grade tumors, according to histology, were classified as low-grade on cytology. Cytological grading is promising and can provide important pre-operative information, but further validation is still needed. To date, this second consensus meeting recommended using the classification system proposed by Camus et al. [76].

The use of cytology to assess lymph nodes for the presence of metastases is recommended as a screening method; however, false negative results may occur, since the punctured area may not comprise, the area affected by neoplastic development [15,52]. Thus, lymphadenectomy and histopathological evaluation of the lymph node in its entirety is always recommended.

### 2.2. Histopathological Analysis

Histopathological grading is considered the main tool to suggest the biological behavior of cutaneous and subcutaneous MCT, and is fundamental in therapeutic decision-making and prognostic investigation [48,78,79,80,81]. A correct histopathological analysis depends on the quality of the collected sample, as well as on good packaging, fixation, and transport practices. It is important to bear in mind that incisional biopsies may not faithfully represent the formation, which may interfere both with the identification of the cutaneous or subcutaneous location, and with the grading and/or identification of criteria and parameters for prognostic evaluation.

Instructions for sample collection and handling are as follows:

1. When it is necessary to perform cytoreductive therapy with the use of corticosteroids or chemotherapy, it is recommended that material first be collected by incisional biopsy, considering that cytoreductive protocols can change the assessment of the cell proliferation index (mitotic count and immunohistochemical analysis for Ki67), and when possible, the excised formation should be reassessed;
2. When an incisional biopsy is necessary, maximum caution is recommended in handling the tumor, in order to avoid degranulation of mast cells and risk to the patient;
3. For a better assessment of the surgical margins, the use of India ink or suture stitches is recommended to identify the lateral and deep margins;
4. When there are multiple nodules, the submission must be completed separately with the appropriate identifications, allowing individual analysis of each lesion;
5. The sentinel lymph node (considered the first lymph node to receive tumor drainage) should be sent for analysis whenever possible, and should be packaged separately to allow for metastasis research [82,83,84].

In terms of margin analysis, performing a trans-surgical pathological analysis to aid in the complete excision of a formation can be considered for MCTs. There are no well-defined, proven, and published criteria for defining free, suspicious, or compromised margins. In addition, it is impossible to differentiate normal mast cells from neoplastic mast cells in most cases. Thus, current interpretations are empirical, although supported by evidence-based medicine, and, thus, require further studies.

It has been suggested to interpret as ‘infiltration’ the presence of mast cells in foci or cords which, when discrete, are considered only if not associated with the cutaneous annex structures and vessels. We interpreted these as suspect, since the skin of allergic dogs tends to present numerous mast cells in the superficial dermis, preferentially concentrated in vessels and annexes. When possible, in these cases, a skin biopsy from a distant location is requested for comparison and better interpretation. Some authors have suggested that low-grade MCTs would not relapse, even when margins are compromised [85], while about 40% of high-grade MCTs would relapse, even when margins are free [86].

**Figure 3:**
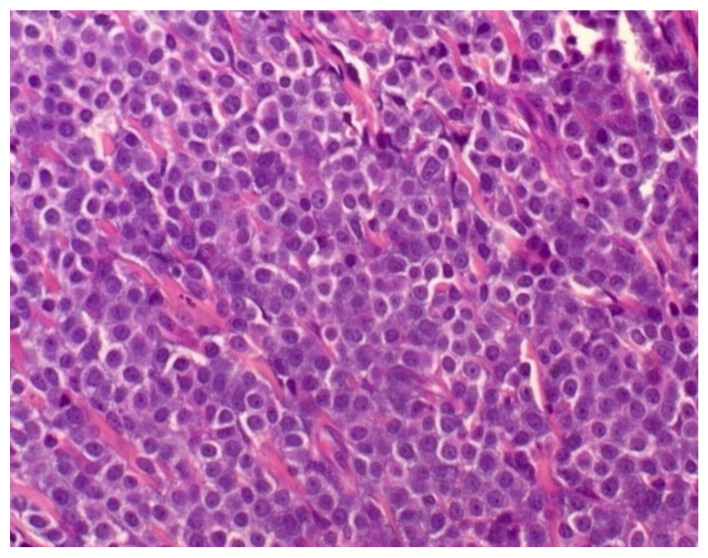
Photomicrograph of a canine mast cell tumor, revealing a round cell proliferation, with evident nucleolus and moderate cytoplasmic granulation. Hematoxylin and eosin staining, Objective: 40×.

The grading system proposed by Patnaik et al. [48] is the most commonly used system for cutaneous histological classification of MCTs, and divides tumors into three groups: grades 1, 2, and 3. It must be emphasized that the Patnaik Grading System should not be used for subcutaneous tumors [64]. According to the authors’ criteria, grade 1 MCT is characterized by rows or clusters of neoplastic mast cells that are well-differentiated and monomorphic, with rounded nuclei, small intracytoplasmic granules, with cell proliferation confined to the dermis. Grade 1 MCTs have no mitotic figures and binucleate cells and may have minimal stromal reactions or necrosis. Grade 2 MCT is characterized by moderately pleomorphic neoplastic mast cells, with round and/or pleomorphic nuclei and intracytoplasmic granulation of varying sizes. They extend deep into the dermis, subcutaneous tissue, and occasionally even deeper. Grade 2 MCTs show zero to two mitosis figures per higher magnification field, discrete areas of edema, necrosis, and collagen hyalinization (Figure 3). Grade 3 MCTs are characterized by neoplastic mast cells with remarkable pleomorphism, rounded pleomorphic vesicular nuclei, containing multiple prominent nucleoli. The cells are arranged in dense layers that replace the subcutaneous tissue and deep planes. Grade 3 MCTs contain three to six mitosis figures per high-power field, and areas of hemorrhage, edema, necrosis, and collagen hyalinization.

Although the Patnaik system is considered the “gold standard” in the prognostic evaluation of canine MCT, the prevalence of cases considered grade 2, and the variability of interpretation between pathologists for the same sample reduce the accuracy of the system. Considering Patnaik’s criteria for grade 3 tumors, when evaluating proliferative indices, some cases with low mitotic indices that present with aggressive behavior are excluded or underestimated [73,79,80,87,88,89]. Shaw et al. [90] evaluated the degree of agreement of the histopathological grading systems in samples collected by incisional techniques (punch, Tru-cut needle and similar devices, and collection with a scalpel) compared to those collected by excisional biopsy, and observed agreement of 96% by the system of Patnaik and 92% by the Kiupel system. They concluded that all techniques were adequate in differentiating between low-grade and high-grade MCTs. Some grade 2/high-grade cases may be underestimated in pre-operative biopsies, and are, thus, not recommended as the only source of therapeutic guidance and tumor staging [54].

To reduce the variability of interpretation and obtain a more reliable histological classification, Kiupel et al. [80] proposed a second classification system for the canine MCTs, using low- and high-grades. When presenting at least one of the following characteristics, the MCT is considered high-grade:

1. A minimum of seven mitotic figures counted in 10 fields of higher magnification, and evaluated in the areas with the greatest number of mitotic figures;
2. At least three multinucleated cells (three or more nuclei) in 10 high-power fields;
3. At least three bizarre nuclei or markedly pleomorphic nuclei in 10 fields of higher magnification; or
4. Karyomegaly [15,80].

In the study by Kiupel et al. (2011) [80], three samples of MCT classified as having a high degree of malignancy did not have the seven mitotic figures counted in 10 fields of highest magnification, which indicates that the number of mitotic figures should not be the only criterion to classify the MCT as being of high degree. There was 100% agreement among pathologists in classifying low and high malignancy MCT [80]. For standardization purposes, it was suggested that the mitosis figures should be counted in the hotspot area.

This consensus guideline proposes the use of both the classification systems suggested by Patnaik et al. [48] and by Kiupel et al. [80], for a better classification of the disease (e.g., “MCT grade 3 according to Patnaik and high-grade according to Kiupel”). The recommendation is in line with the recommendation of the American College of Veterinary Pathologists and the Veterinary Cancer Society [54].

The evaluation of the mitotic count also assists in MCT prognostic investigation. The mitotic count must be evaluated in an area of 2.37 mm^2^, avoiding the use of 10 fields with high magnification (400×). Alternatively, the field size must be described, as well as the correction and equivalence to the reference work used, since differences between analyzed microscopic areas, which vary with the field number of the microscope eyepiece used, can reach up to 400% between one device and another. The importance of correctly applied methodology for defining a variable of prognostic value cannot be neglected [91]: only then can the cutoff values suggested in the literature be validated. The mitotic count has been described as a prognostic factor independent of other histopathological features, having higher indices associated with a worse prognosis [92,93]. Reported cutoff values for the number of mitotic figures counted in 10 fields of highest magnification can range from 5 to 10 [78,92,93]. Until other prospective studies define statistically calculated cutoff values for MCTs, the authors of this consensus guideline recommend using the methodology of Romansik et al. [92] for classifying the index into ≤5 and >5 mitotic figures, or of Elston et al. [93], for ranking the index at 0; 1–7, and >7 mitotic figures. Elevated mitotic indices (>5 mitotic figures or >7 mitotic figures) are related to worse prognosis.

Most veterinary pathologists agree that the mitotic count is an important element for grading canine cutaneous mast cell tumors and the current approach is to determine the mitotic count in 10 consecutive high-power fields with the highest mitotic activity. However, there is variability in area selection between pathologists. Bertram et al. [94,95] have used computerized calculation of mitotic counts in mast cell tumors and verified that the mitotic count is area dependent, and the assistance of a computerized system may improve the interobserver reproducibility and accuracy. Therefore, deep learning techniques aim to help, but not replace, the pathologist evaluation in MCTs. 

Considering subcutaneous MCTs, recent studies have sought to trace specific prognostic factors, although these are mostly intermediate to low-grade tumors [84,96,97]. Thompson et al. [84] classified subcutaneous MCTs into three patterns:

1. Circumscribed;
2. Combined (infiltrative/circumscribed); and
3. Infiltrative.

In this study, cases with lower survival were associated with a mitotic count > 4 in 10 fields of high magnification (40×), an infiltrative histological pattern, and the presence of multinucleated neoplastic mast cells. It is also recommended to send a sample, including the associated cutaneous portion, for analysis, for the correct classification of MCTs in the subcutaneous tissue. This consensus recommends the classification by Thompson et al. [84] for the classification of subcutaneous MCTs.

For lymph node histopathological evaluation, it is recommended that serial sections of the lymph node be made, transverse to the longest axis, and with each section 2 mm-thick, in order to represent the largest possible area of the subcapsular sinuses and lymph node parenchyma, with special attention to capsular and extra-capsular invasion. Immunohistochemical analysis and the toluidine blue staining technique can be added for a more effective diagnosis. For classification, Weishaar et al. [98] proposed a model that divides cases into: HN0 (non-metastatic lymph node), characterized by absent or rare mast cells (0–3), isolated in the sinuses (subcapsular, paracortical, or medullary), and/or lymph node parenchyma per 40× field. HN1 (pre-metastatic lymph node), characterized by more than three isolated mast cells in the sinuses (subcapsular, paracortical, or medullary), and/or lymph node parenchyma per 40× field, in at least four fields. HN2 (early or early metastasis), characterized by aggregates (clusters) of mast cells (more than three cells) in the sinuses (subcapsular, paracortical or medullary) and/or parenchyma, or sinusoidal cords of mast cells. HN3 (obvious or advanced metastasis), characterized by altered lymph node architecture, with foci of mast cells organized in a mantle or cord. However, the authors of this consensus have reservations about the term “pre-metastatic” used in the HN1 classification, as it can generate doubts, and should not be confused with cases of already established metastasis.

Histochemical techniques, such as Giemsa, toluidine blue, and Alcian blue-safranin are extremely important to establish a differential diagnosis with other round-cell neoplasms, and to identify intracytoplasmic granulation, particularly in cases where cytoplasmic granules are scarce. However, poorly differentiated MCT cases may require immunohistochemistry for diagnostic confirmation [99].

In human medicine, the tissue freezing technique is typically used to determine the presence of tumor cells in surgical margins and metastases in sentinel lymph nodes [100,101]. Its accuracy is similar to that of tissues embedded in paraffin [102,103], and this technique can be perforMed. to confirm the diagnosis of MCTs, as well as to analyze the lateral surgical margins and depth of the tumor during surgery. Historically, there are several stains that can be used to detect mast cells in histology; however, in frozen tissues, toluidine blue remains the stain of choice [104]. It is suggested that histopathology perforMed. by freezing MCTs should be used as a diagnostic tool, and to assess the surgical margins. Histological grades currently used should not be applied when using the freezing technique; rather, histopathology of paraffin-embedded tissues is necessary. Sentinel lymph nodes should not be frozen, but should be preserved for histopathological grading according to Weishaar et al. [98].

### 2.3. Immunohistochemistry and Histochemical Staining Methods

Several studies have used immunohistochemistry to analyze markers that aid in the differential diagnosis and understanding of the biological behavior of MCT; however, few antibodies have shown predictive or prognostic value [83]. The most widely used immunohistochemical markers in the prognostic evaluation of MCT, and that are recommended by the authors of this consensus, are Ki-67 (proliferative index) and *KIT* [32,79,105,106,107,108,109,110].

**Figure 4:**
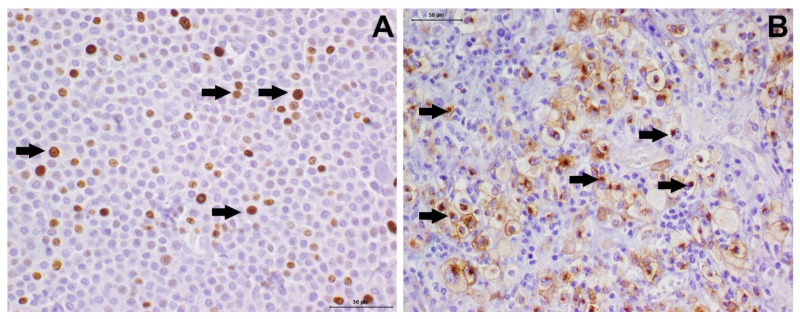
Ki67 and KIT immunoexpression in canine mast cell tumors. (**A**) Ki67 expression in a mast cell tumor showing more than 23 positive cells (arrows–brown staining). (**B**) KIT immunohistochemistry revealing pattern II (focal cytoplasmic–arrows). Harris hematoxylin counterstaining.

Ki-67 is a proliferative marker. The methods used for its calculation vary across studies [32,105,107,108,109], which can generate variation in interpretations and observations. Thus, this consensus recommends using the methodology proposed by Webster et al. [32] to assess Ki-67 staining in cases of cutaneous MCTs, and the methodology described by Thompson et al. [84] for subcutaneous MCTs. To interpret the labeling in cutaneous MCTs, a Ki-67 value > 23 positive cells in five fields of higher magnification is used (Figure 4A), and is associated with higher risks of recurrence and metastasis development [32].

For KIT, studies have determined three patterns of protein expression in canine MCTs: the membranous pattern (KIT pattern 1), focal cytoplasmic pattern (KIT pattern 2) (Figure 4B), and diffuse cytoplasmic pattern (KIT pattern 3) [79,106]. The different patterns correlate with tumor aggressiveness, with focal or diffuse cytoplasmic patterns (KIT patterns 2 and 3) being associated with a poor prognosis [106]. However, recent studies have shown that only KIT pattern 3 correlated with a worse prognosis [11]. It is recommended that immunohistochemical reports use the nomenclature determined by Kiupel et al. [106], aiming at standardization of sample analysis. However, different studies have used different antibodies, at different concentrations, and using different protocols, which make it difficult to make comparisons. Considering the prognostic and predictive potential of both markers, the authors of this consensus strongly recommend a standardization of the technique used, following the criteria proposed by Kiupel et al. [106].

Considering the possibility of other KIT activation mechanisms, recent studies have evaluated the measurement of phosphorylated KIT (pKIT) as a possible prognostic marker [111,112]. A study by Thamm et al. [112] found that the immunohistochemical detection of pKIT in patients with MCTs may predict prognosis and biological behavior. Further studies are needed to validate this test for routine inclusion. Immunohistochemistry should be used after histopathological analysis, in cases of inconclusive initial diagnosis, or to add prognostic and predictive information in cases with a confirMed. diagnosis, to assist in the definition of adjuvant therapies.

Webster et al., 2007 [32], compared three proliferation markers for the assessment of cellular proliferation. The markers were Ki67, the proliferating cell nuclear antigen (PCNA), and the argyrophilic nucleolar organizing region (AgNOR). The study revealed that increased Ki67 and AgNOR counts were both associated with significantly decreased survival. Authors indicated that AgNOR, PCNA, and Ki67 staining provides mutually exclusive and complementary information, and that they could be used together to inform more precisely about the cell cycle than when they are used independently. 

AgNOR is a silver-based staining that can be easily perforMed. in FFPR whose advantage is the cost, since it is cheaper than immunohistochemistry. However, there are interobserver variabilities, which must be taken into account. In addition, more recently Smith et al., 2017 [113] aiMed. to determine if the extent of surgical excision affected recurrence rate in dogs with grade II MCT with low proliferation activity. The authors suggested combined AgNORxKi67 (Ag67) values to determine low cell proliferation in grade 2 MCTs. 

### 2.4. KIT Mutation

*KIT* encodes the receptor tyrosine kinase KIT, a membrane receptor with tyrosine kinase activity for SCF, which stimulates mast cell growth. In 1999, two studies described the presence of mutations in *KIT* in canine MCTs, after observation of an ITD [28,33]. The KIT protein is composed of an extracellular domain (coded by exons 1–9), a transmembrane portion (exon 10), juxtamembrane (exons 11 and 12), cytoplasmic (exon 13), and phosphotransferase lobes (exon 17) [39]. Mutations in extracellular domains are called regulatory-type mutations, while mutations in intracellular domains (exons 13–21) are called enzymatic-type mutations [114].

When SCF binds to the KIT receptor, the cytoplasmic portion of the receptor undergoes autophosphorylation. In the presence of the ITD in exon 11, the receptor is phosphorylated, irrespective of whether SCF is bound. pKIT activates signaling pathways that stimulate neoplastic mast cell growth [28]. Therefore, the presence of this mutation is directly responsible for the uncontrolled proliferation of the tumor, which, in this case, presents a worse prognosis [32]. The discovery of an aggressive behavior of MCT by a constitutive activation and signaling of a tyrosine kinase receptor has resulted in research into the use of TKIs in MCTs [38,115].

About 30% of canine MCTs have ITDs in the juxtamembrane (exons 11 and 12) and extracellular (exons 8 and 9) domains of the *KIT* [37]. ITDs located in exon 11 represent 60–74% of all mutations and are associated with a worse prognosis and greater chances of recurrence and metastasis [11,30,32,40,42]. The prognostic value of detecting mutations in other exons of *KIT* has not been systematically investigated. Brocks et al. [42,43] compared cases of MCTs with mutations in exon 8 and exon 11 and observed that the presence of ITDs in exon 8 were associated with a more favorable prognosis.

Other mutations, deletions, and insertions have also been found in this tumor type, but are rarely observed [11,28,30,32,33,34,35,36,37,38,116,117,118,119]. Only two studies evaluated the presence of mutation in *KIT* in subcutaneous MCTs, with a negative result in all cases [84,111]. Mutation analysis of *KIT* (most commonly for exon 11 ITD) is offered by several laboratories and is based on polymerase chain reaction tests. Furthermore, such analyses should be included in a panel of markers for canine MCT, as they have prognostic value [120]. These tests must discriminate for which mutation, deletion, or insertion polymorphisms should be searched, as many of them do not lead to constitutive phosphorylation of the KIT [32,37,40,115,120].

### 2.5. Staging

Staging should always be perforMed. with the objective of defining the extent of the disease, which directly influences therapeutic decision-making and prognosis [15,52]. The most widely practiced staging involves lymph node evaluation, abdominal ultrasound, and chest radiography. However, Book et al. [121] found that the sensitivity of ultrasound to detect splenic and liver metastases was 43% and 0%, respectively, suggesting that fine-needle aspiration cytology or guided biopsy should be perforMed. to obtain complete staging. However, in clinical routine, the puncture of these organs cannot always be performed. Warland and colleagues [122] also suggested that chest radiography would have less importance in the staging of canine MCT, and is not mandatory for its performance. However, it is important to remember that, despite being low, the possibility of pulmonary dissemination still exists in cases of MCT.

Computed tomography (CT) is an imaging technique that has been used more frequently in recent years as a more sensitive tool in identifying metastatic lesions in different neoplasms. Hughes et al. [123] evaluated CT and spleen and liver cytology results of dogs with MCT and observed that, in most dogs with cytology positive for metastases in the spleen or liver, these organs had a normal appearance on CT, and, therefore, they concluded that the tool was not effective in identifying early forms of metastatic lesions. Dogs with positive cytological evidence of mast cell infiltration in the spleen, liver, or both have a worse prognosis [121].

The identification of metastases to the lymphatic system plays an important role in the staging of MCTs. Sentinel lymph nodes, considered the first lymph nodes in the lymphatic chain to drain the tumor, are important in the staging of several types of human cancer, providing important prognostic information. In veterinary medicine, evaluation of the sentinel lymph node during staging should always be incorporated in malignant neoplasms of lymphatic dissemination, such as carcinomas, melanomas, and MCTs [124].

Krick et al. [125] demonstrated that cytopathological evaluation of lymph nodes from dogs with MCTs provides valuable clinical information and correlates with tumor grade and prognosis, in addition to being a practical and non-invasive technique. However, cytopathological analysis of lymph nodes has a sensitivity ranging from 68% to 75%, which may generate conflicting results, suggesting that histopathological examination of locoregional lymph nodes should ideally be perforMed. [126,127]. Warland et al. [122] observed that no tumor had distant metastases without affecting the sentinel lymph nodes, suggesting less importance of searching for metastases in distant organs when lymph node metastasis is absent.

The identification of the sentinel lymph node in clinical routine remains challenging, and it is important to emphasize that locoregional lymph nodes, those in anatomical proximity to the tumor, may differ from the sentinel lymph nodes, due to lymphatic drainage changes promoted by the tumor [128,129]. In the study by Worley [128], lymphatic mapping of dogs with MCT was performed, and it was observed that the sentinel lymph nodes did not correspond to the locoregional lymph node in 42% of cases. Aiming to increase the accuracy of sentinel lymph node detection, Fournier et al. [130] evaluated the effectiveness of contrast ultrasound in identifying these lymph nodes, showing accurate detection in 95.2% of cases. However, further studies are still needed to validate this technique. 

Regarding tumor staging, different clinical staging systems have been proposed for canine MCT. The World Health Organization (WHO) proposed a clinical staging system (Table 1) for canine MCT, which classifies dogs with the presence of stage II lymph node metastasis and dogs with the presence of multiple tumors as stage III, even with lymph nodes free from metastasis. This classification is questioned by several studies, particularly in terms of MCT grade 3 [15,20,52]. 

Another staging system for MCTs was proposed during the Southern European Veterinary Conference (SEVC) in Barcelona in 2008 [52], considering as stage IV any patient with metastatic lymph node disease, regardless of the number and size of the formations (Table 2).

Given the practical divergences of current staging systems, Horta et al. [11] proposed an improvement in the staging system already established by the WHO, allowing classification of dogs with MCT according to the risk of recurrence and metastasis. The study demonstrated that patients with multiple lesions without involvement of regional lymph nodes generally have a better prognosis compared to those with a single lesion, but with lymph node involvement. Thus, in the new proposed staging, the presence of metastasis in a regional lymph node would classify the patient as stage III. Stage IV would be characterized by the presence of extensive and infiltrative formations or multiple formations with lymph node involvement, and stage V, when there is the presence of distant metastases (Table 3).

Given the challenges in the practical applicability of the staging systems proposed to date, and considering the variable biological behavior of the disease, it is still not possible to standardize a single system for cutaneous MCTs in dogs. Interestingly, other characteristics, such as race and tumor location, are negative prognostic factors that are well established in the literature, and that are not yet considered in current staging systems [11,15,131]. Thus, further studies are needed to validate the staging already proposed or even to propose new adaptations.

## 3. Prognostic Factors

The biological behavior of canine MCT is variable. These MCTs may manifest a low metastatic potential or could show extremely aggressive behavior, leading to metastasis and death. These factors must be evaluated in conjunction with the particular features of each patient and can be used as a tool to anticipate the biological behavior of the tumor, and to guide the treatment [132]. London and Thamm, 2013 [133], presented a useful list of prognostic factors for canine MCTs.

Horta et al. [11] studied 149 cases of canine MCTs. Their multivariate analysis showed a higher risk of death (62%) related to MCT in patients with tumor recurrences (local or distant) and lymph node metastasis. In the absence of these characteristics, other prognostic factors proved to be reliable predictors of an intermediate risk of death related to MCT (14%). In this context, the histological grading, mitotic count, a high expression of Ki-67, KIT pattern 3, and ITD in exon 11 of *KIT* stood out. In the absence of any of these factors, MCT-related risk of death (4%) was reduced. Moore et al. [55] suggested that some dogs with high-grade solitary MCTs (Kiupel criteria) may have a favorable prognosis, particularly those tumors of a smaller size and lower mitotic count. Thus, they concluded that clinical staging and mitotic count can be as useful as histological grading for obtaining prognostic information, although it is essential that they be interpreted together. Factors, such as tumor size and presence of lymph node metastasis, both included in clinical staging, are considered important prognostic factors, directly influencing the survival obtained with different treatments [133,134,135,136]. 

As previously mentioned, histological grading is currently the most reliable prognostic factor for MCTs. Although some studies found an association of the Kiupel classification with a higher prognostic value [63,137], it is still recommended that both classification systems be used [15,138]. However, even with the association of both histological classifications, a percentage of MCTs still manifest unpredictable biological behavior, regardless of grading [11,112]. Stefanello et al. [131], emphasized the importance of evaluating the histological grade together with other clinical factors of prognostic relevance, such as tumor size, anatomical location, and the presence of metastases. In addition, other factors that have previously been associated with a worse prognosis also include the presence of multiple lesions, erythema, pruritus, tumor ulceration, and the presence of systemic clinical signs [15].

Given the variable behavior of canine MCTs, there is a need to include new tests that provide additional information on biological behavior and clinical prognosis. Immunohistochemical markers of cell proliferation and differentiation are widely studied and mainly include Ki-67 immunostaining and KIT receptor localization [11,32,108,109,139,140]. However, some studies have investigated the real prognostic value of these immunomarkers. Costa-Casagrande et al. [141] evaluated KIT immunostaining patterns and found no prognostic correlation. Similarly, in the study by Horta et al. [11], Ki-67 levels and the KIT expression pattern were not particularly informative or relevant for prognosis. Furthermore, in this study, the presence of mutations in *KIT*, particularly in exon 11, had a significant prognostic impact and was not correlated with aberrant expression of KIT. In an attempt to obtain more robust prognostic information, Ki-67/KIT double-staining was previously evaluated, and was shown to be valuable for prognosis [142].

Other immunomarkers have also been studied in recent years, including vascular endothelial growth factor (VEGF), tumor suppressor gene TP53, cell nuclear antigen (PCNA), argyrophilic organizer regions (AgNORs), and apoptotic proteins (BAX, BCL-2) [41,138,140,141,143,144,145]. In the study by Vascellari et al. [140] the expression of Ki-67, BCL-2, and COX-2 were evaluated for their possible prognostic values. They observed that expression of Ki-67 and mitotic count were associated with histological grade and survival, while the expression of BCL-2 and COX-2 showed no significant association with prognosis. In contrast, Barra et al. [144] demonstrated that high expression levels of BCL-2 and BAX were associated with a worse prognosis.

Bergman et al. [143] investigated the Ki-67, PCNA, and AgNOR expression and identified its positive association with histological grade. However, in the study by Scase and colleagues [107], although AgNOR and Ki-67 levels had prognostic value, PCNA expression showed no correlation with prognosis. Freytag et al. [146] carried out an extensive review and meta-analysis aiming to identify the immunohistochemical markers that have the greatest prognostic value in this context to date. They observed that Ki-67 and KIT expression have prognostic value, and the pro-apoptotic immunomarker BAX also showed promising application as a prognostic factor in MCTs. 

Regarding prognostic factors of canine subcutaneous MCTs, Thompson et al. [84] found that MCTs with Ki-67 > 21.8 and mitotic count > 4 were more likely to relapse and metastasize. Subsequently, Thompson et al. [111] correlated the expression of KIT, VEGFR2, and the presence of mutations in *KIT* with the prognosis of subcutaneous MCTs. They found that pKIT, aberrant KIT localization, and VEGFR2 expression were associated with shorter survival times, disease-free duration, and increased metastatic rate. Similarly, Gill et al. [97] evaluated negative prognostic factors in subcutaneous MCTs, reporting that proliferation indices, particularly AgNORs and mitotic count, are useful for predicting the biological behavior of this type of presentation. Silva et al. [147] investigated the prognostic value of VEGF-A, VEGFR2, pVEGFR2, VEGF co-receptor Neuropyla-1 (NRP-1), and c-Cbl-1 in a group of canine subcutaneous MCTs associated with patient survival. High expression of VEGFR2, pVEGFR2, and c-Cbl-1 was shown to correlate with the disease-free interval, indicating the potential prognosis of these markers. However, there is a need to establish more routine immunohistochemical markers for subcutaneous MCTs, since the identification of KIT expression and mutations in its receptor have not been validated as prognostic factors. Currently, the main validated prognostic factors for subcutaneous MCTs should be considered to be: (1) mitotic count > 4; (2) presence of multinucleation; and (3) infiltrative growth pattern [81].

Given the results of previous studies, it can be inferred that there is no single technique or factor that can unambiguously determine the behavior of cutaneous MCTs. Thus, the authors of this consensus statement recommended the combined assessment of different factors, including clinical staging, history of tumor recurrence, histopathological grade (Patnaik and Kiupel gradations and mitotic count) and molecular data, highlighting the assessment of Ki-67 level, expression of KIT, and detection of mutations in *KIT*, mainly in intermediate- to high-risk cutaneous MCTs [148]. The most studied and validated prognostic factors are summarized in Table 4.

## 4. Therapeutic Approach

To define the best therapeutic approach, all the points raised during the diagnostic investigation should be considered, to determine the ideal approach for each individual case.

### 4.1. Local Treatments

#### 4.1.1. Surgery

The main therapeutic modality indicated in cases of canine MCT is surgical excision, which should be perforMed. whenever possible, according to the patient’s clinical conditions and staging [15,149]. Previously, wide excision was advocated, regardless of histopathological grading and associated prognostic factors, with application of a 3 cm lateral margin and a fascial layer for the deep margin. However, recent studies have indicated different approaches related to specific prognostic factors, mainly clinical staging and histopathological grading.

The application of proportional margins was initially suggested as a conservative alternative for dogs affected by cutaneous MCT, in their first surgical intervention, and after complete staging. The concept initially consisted of using a lateral margin proportional to the largest tumor diameter, for tumors below 4 cm, and a fixed margin of 4 cm for tumors above this diameter, maintaining a deep margin of at least one fascial plane [150]. Later, the concept was adapted, with a 2 cm lateral and a deep safety margin of a fascial plane being recommended for grade 1 or 2 tumors, and up to 4 cm in diameter, providing effective local control of the disease [151,152,153,154], with a recurrence rate of 0–4% [82,150,155]. In cases of high-grade (or grade 3) MCTs, there is a high recurrence rate (36%) regardless of the surgical margins used, as observed in the study by Donelly et al. [156].

Associated lymphadenectomy is recommended during initial surgical intervention. Ferrari et al. [127] demonstrated that approximately half of the patients who did not present alterations on palpation already had metastases when histopathological evaluation was performed. When tumor removal is perforMed. before lymph node removal, it is recommended to change the material and surgical drapes, to avoid tumor implantation [133]. Lymphatic mapping by injection of radionucleotides or radiographic contrasts is indicated to aid in the characterization of the sentinel lymph node, whose trans-surgical identification can be facilitated by the use of vital dyes, such as patent blue [157]. Regional lymphadenectomy, in cases of metastatic lymph nodes, has therapeutic potential, with evidence of reduced local recurrence and the development of distant metastasis [158]. Lymphadenectomy, when perforMed. in intracavitary sentinel lymph nodes, can complicate the surgical procedure. Thus, the risk and benefit must be evaluated in each case.

When the lesion is located in the scrotum, orchiectomy, and testicular pouch ablation are performed, and when the foreskin is involved, penectomy associated with urethrostomy is recommended [159]. In the limbs, amputation or tumor exeresis, associated with reconstructive techniques such as subdermal or axial flaps of advancement, rotation, transposition, and interpolation, or free grafts, can be considered in order to preserve the limb [15,52,159]. Second intention healing can be perforMed. when there is no possibility of applying reconstructive techniques with adequate repair of the surgical lesion. Second intention healing is preferable in the face of compromised margins [52,159].

Regardless of the technique used, it is necessary to avoid excessive manipulation of the lesion, seeking to avoid stimulation of mast cell degranulation and its consequent adverse effects [159]. In cases of tumor recurrence after initial excision, the main associated factor tends to be histological grading, with low recurrence rates after excision of grade 1 or II MCTs [160,161].

#### 4.1.2. Electrochemotherapy

Electrochemotherapy (ECT) has emerged as a viable technique at the experimental level for the treatment of superficial and mucosal tumors in the 1990s [162]. In November 2006, the technique was standardized in the European community for use in humans. The technique combines the intratumoral and/or intravenous application of specific drugs, followed by the application of electrical pulses, leading to temporary electropermeabilization of the cell membrane, with the objective of maximizing the entry of the chemotherapeutic agent to enhance its cytotoxic effect [163,164]. In general, to perform this technique, the main drugs used are bleomycin and cisplatin, both with intratumoral use, but only bleomycin for intravenous use [165]. The use of conductive gel can facilitate the efficiency of the technique by improving the distribution of the electric field in the treated region [166,167]. In addition to direct cytotoxic effects, ECT also has indirect antitumor effects through action on the tumor vasculature and local immunity. Vascular effects lead to vasoconstriction and destruction of the local vasculature, and immunological effects lead to signaling for immunogenic cell death [165,168,169].

ECT in MCTs can be used as a single therapy, associated with the trans-operative period, or as an adjuvant therapy to surgery when margins are compromised [170]. Spugnini et al. [171] used ECT with intralesional bleomycin (1.5 UI per mL) in cases of canine MCT with compromised surgical margins, and observed that, in 85% of cases, the mean time to recurrence was 52.7 months. Kodre et al. [172] reported ECT with intralesional cisplatin (1 mg/cm^3^ of tumor) as the only therapy to treat MCT and obtained recovery rates similar to those obtained after surgical excision, without associated chemotherapy and lesion regression for a mean of 31.5 months. Spugnini et al. [173] evaluated the effect of ECT with intralesional cisplatin in canines with grade 2 and 3 MCT (relapsing or not) or with compromised margins, and showed a 78% response rate without major adverse effects. 

Lowe et al. [174] evaluated the effects of different applications of ECT with IV bleomycin (15 IU per m^2^) in dogs with MCT, being applied as a single therapy trans-surgically, as an adjuvant to surgery, and in recurrent MCTs. The best results were obtained in the adjuvant to surgery group and in the trans-surgical group (93% and 91% complete response [CR], respectively), followed by the single therapy group (80% CR), and finally, the relapse group, where ECT was applied after local recurrence, at the site where the surgery was perforMed. (64% CR). The group where ECT was used in the trans-surgical period had the longest disease-free interval. Adverse effects, such as degranulation, were reported in two of 28 cases [171]. Kodre et al. [172] stated that the vasoconstriction caused by electrical pulses prevents the release of inflammatory mediators contained in the mast cell granules into the bloodstream, with no adverse effects being observed in 12 patients treated in their study. The mean size of the tumors treated in their study was 2.9 cm^3^, with greater efficacy being observed in tumors < 2 cm. Thus, to prevent the effects of mast cell degranulation, it is important to use a concentric approach, through the application of electrical pulses initially at the periphery of the tumor and moving towards the center. The use of prior antihistamines is recommended in association with any manipulation of MCTs, including for performing ECT, particularly those with larger diameters.

ECT is an easy, effective, and safe local treatment for MCT cases and can be an alternative treatment to surgery, specifically for smaller nodules in which a long-lasting CR can be achieved with a single treatment session, or when the nodule cannot be resected due to its location. ECT can also be combined with surgery for larger lesions without significant toxicity, being applied in the surgical bed, after tumor resection, particularly for MCTs where margins may not be reached due to tumor extension or location. Post-surgery, ECT can be applied in MCTs with incomplete resection (compromised or small margins), with the application of therapy being indicated at 2–4 weeks after surgery. Preliminary studies using ECT combined with gene transfer by electroporation of canine IL-12 (electro gene transfer) have shown promising results for the treatment of MCTs [173,174,175,176].

Thus, the authors of this consensus meeting recommend the use of ECT in specific cases of canine MCT: (1) as a single therapy in small tumors < 3 cm that are difficult to resect surgically either because of the location or the number of tumors (multiple MCTs); (2) intra-operatively, in cases where it is difficult to obtain free margins due to tumor extension or location; (3) as an alternative treatment for cases with compromised or small margins.

#### 4.1.3. Radiotherapy

Radiotherapy in dogs with MCT can be perforMed. with definitive protocols (consisting of 12–19 fractions perforMed. 3–5 times a week), prior to surgery, or post-operatively in cases of incomplete resection, or with palliative protocols (3–6 fractions perforMed. once a week) in order to reduce tumor volume partially and control pain [177]. In cases where the diffuse and/or extensive nature of the tumor mass precludes surgery, radiotherapy should be considered, although studies on the use of radiation as a sole therapy to treat canine MCT are scarce [178]. The tumor response to radiation is considered to be dose-dependent; thus, increasing the dose can lead to a significant increase in tumor response [178].

Adjuvant radiotherapy, in a definitive protocol, was used to treat grade 2 MCTs in cases where complete surgical resection was not possible, and proved to be effective and safe, with significant improvement in local disease control [179,180,181,182,183]. In a study of dogs with grade 2 MCTs, the radiotherapy protocol was 18 daily fractions of 3 gray (Gy) for a total of 54 Gy. This study showed that 94% of patients were in remission within 1 year and 88% within 5 years [179]. The use of this therapy has also been indicated in incompletely excised grade 3 MCTs, with tumor control times and median survival of 17 and 20 months, respectively [134]. The MCTs located in the head and in the distal region of the limbs are important examples of sites in which it is difficult to remove the tumor completely while preserving the local anatomy; therefore, in these cases, radiotherapy is indicated [178].

For best results regarding disease-free intervals, irradiation directly into the primary tumor or surgical scar (with a 3 cm safety margin) and into regional lymph nodes is recommended [178,184,185]. The inclusion of regional lymph nodes in the field of radiotherapy, whether palpable or not, is extremely important for tumor control [182,184,185,186]. In a study of dogs with high-grade MCT treated with definitive radiotherapy, prophylactic treatment of regional lymph nodes significantly increased the time to tumor control (>2381 days) as compared to cases where the lymph nodes were not treated (197 days) [186].

A study in dogs with unresectable MCTs of varying degrees used a hypofractionated radiotherapy protocol (4 fractions of 8 Gy) at 7 day intervals and reported an 85% partial or complete response rate [178]. Carlsten et al. [187] perforMed. a prospective multicenter study with a combination of hypofractionated radiation treatment, toceranib, and prednisone for measurable canine MCT and concluded that this protocol is a viable treatment option for unresectable MCTs, is well-tolerated and effective in most dogs, with response rates and durations higher than those reported for toceranib as a single treatment agent for MCT. Stiborova et al. [188] evaluated the toxicity related to the combination of radiotherapy with vinblastine chemotherapy and concluded that the combination is safe and did not increase chemotherapy-related myelosuppression as a single treatment.

Acute side effects resulting from radiotherapy include erythema and epilation in the treated area, which start from the second week of treatment. Alopecia, hyperpigmentation, and thickening of the skin in the affected area can also be seen as late effects [178]. In a study using hypofractionated radiotherapy, many animals developed mild erythema in the treated area, without severe signs related to degranulation and histamine release.

Although still inaccessible in Brazil, radiotherapy proves to be effective as an auxiliary tool in the treatment and control of canine MCTs, particularly in cases where surgical therapy is difficult or incomplete.

#### 4.1.4. Tigilanol Tiglate (Stelfonta®)

Tigilanol tiglate (TT) is a natural product extracted from the seed of the *Fontainea picrosperma* (Blushwood, from the family Euphorbiaceae) native to Australia and purified by high pressure liquid chromatography. It is a well-characterized small, diterpene ester molecule of the tigliane class [154,155]. The composition of Stelfonta® is 1 mg TT per mL, prepared in 40% propylene glycol, 30 mM acetate buffer, pH 4.2. It must be stored strictly at a temperature of 2–8 °C.

Its indication for use is in dogs with cytological diagnosis of cutaneous MCT (in any region) or subcutaneous MCT (in the thoracic limbs, below the elbow, or the pelvic limbs, below the tibiotarsal joint), at different stages, but without involvement of regional lymph nodes based on palpation and lymph node cytology if it is palpable. For use, the dog must have been without treatment for at least 3 months. The lesion must have a diameter greater than 1 cm, and a volume ≤ 10 cm^3^, without ulceration, so that the drug does not escape after its application. TT causes vascular rupture and acute local inflammatory response within 4 h, followed by necrosis within 1–4 days and tumor destruction for a period of 4–7 days, with subsequent resolution of the wound within a period varying from 28 to 35 days [189,190,191,192].

TT is applied intratumorally and radially, at a dose of 0.5 mL per cm^3^ of tumor volume. Tumor volume is determined using the modified volume calculation for ellipsoid structures = 1/2 × length × width × depth (cm). Prednisone must be used before (0.5 mg/kg, every 12 h, for 2 days) and after (0.5 mg per kg every 24 h, for 3 days) the application, in addition to the use of promethazine (0.5–1.0 mg per kg every 12 h for 7 days); and ranitidine (2.0 mg per Kg, every 12 h for 7 days). When total tumor remission does not occur, TT can be reapplied after 28 days, although the volume must be calculated again [190,191,192,193,194].

TT has been the subject of studies mainly in the United States and Europe, with positive results, tolerable toxicity, ease of administration, and being capable of promoting adequate local tumor control [188,189,190,191,192,193]. The application of TT in felines is contraindicated. Up to the time of writing, TT is not available for routine use in Brazil.

### 4.2. Systemic Treatments

#### 4.2.1. Anticancer Chemotherapy

Systemic therapies used adjuvant to surgical treatment are indicated in specific cases, which present an intermediate to high risk of death related to cutaneous MCT, particularly those in advanced stage, grade 3–high grade tumors with a high mitotic count. Although histopathological grading is considered to be the most reliable criterion of prognostic value in MCTs, other prognostic factors must be considered when making chemotherapy decisions, such as the proliferative activity, tumor location, and presence of multiple tumors [15]. 

The use of chemotherapy can also be indicated in the preoperative period, terMed. neoadjuvant chemotherapy, used in cases of large tumors or located in anatomical sites that are difficult to approach surgically, aiming at controlling disease progression in the perioperative period and reducing its tumor dimensions, allowing for more adequate local control [67,195]. However, the reduction in surgical margins based on tumor shrinkage after debulking is a controversial topic in the current literature. Stanclift and Gilson [196], used prednisolone for tumor debulking and concluded that the adoption of 3 cm lateral surgical margins based on tumor size after neoadjuvant treatment were sufficient to obtain free margins. However, it is known that microscopic reduction may not follow macroscopic tumor reduction, and may not occur proportionally. Thus, further studies are needed to determine the adequate extension of the surgical margins necessary after debulking. Importantly, the response to neoadjuvant chemotherapy treatment tends to be temporary, with an average duration of 40–70 days, with surgical intervention being recommended during this period [15,52]. 

Chemotherapy, with or without other treatment modalities, is also indicated for advanced and/or inoperable disease, whether due to tumor dimensions, the number of lesions, or the presence of metastases, thus aiming at palliative control, disease stabilization, and maintenance of quality of life [197,198]. Multidrug therapy protocols promoted a greater response than single-agent chemotherapy [199]. However, simple protocols with a single chemotherapeutic agent, usually associated with prednisone or prednisolone, have become more popular. Sequential association of these protocols in an adjuvant modality demonstrated increased disease-free interval and survival in high-risk dogs [200]. Numerous protocols have been described in the literature; however, data on disease-free interval and other important criteria, to compare their effectiveness, are scarce [197]. It is known that MCTs have different response rates to different chemotherapy protocols and the occurrence of adverse effects may be common among patients [139].

In most studies, vinblastine is considered to be the first-line therapy for treating canine cutaneous MCT, using a dose of 2.0 mg/m^2^ every 1 or 2 weeks, with or without prednisone [135,201,202,203]. Generally, the therapeutic dosage is defined as close to the maximum tolerated dose, in order to optimize the cytotoxic effect on tumor cells. In dogs, the dose-limiting toxicity of vinblastine is characterized by neutropenia, with its nadir occurring approximately 7 days after administration [201,202,203,204,205].

Webster et al. [139] described that treatment with vinblastine at a dose of 2.0 mg/m^2^ once a week for 4 weeks, and prednisone at a dose of 2.0 mg/kg daily, after surgery, was beneficial for dogs with grade 3 MCT, when compared to those treated with surgery alone. Furthermore, dogs with *KIT* mutations that were treated with this protocol had a longer disease-free interval and survival duration. Thamm et al. [203] evaluated the responses of 61 dogs with high-grade MCT treated with complete excision and with vinblastine as adjuvant treatment at a dose of 2.0 mg per m^2^, every 7 or 14 days; and prednisone at a dose of 2.0 mg per kg, with a mean overall survival time of 1374 days. In contrast, Rungsipipat et al. [206] observed a partial response rate of 78.2% in dogs with grade 3 MCT who were treated with vinblastine at a dose of 2.0 mg per m^2^ in weeks 1, 2, 3, 4, 6, 8, 10, and 12, and prednisolone at a dose of 1.0 mg per kg for 4 weeks and then 0.5 mg per kg for 8 weeks.

In the last decade, several studies have sought to optimize the dosage and effectiveness of treatment with vinblastine. It was found that vinblastine can be safely administered at a dose of 3.0 mg per m^2^, but that hematological and gastrointestinal effects can be observed above this level [55,198,207,208]. Vickery et al. [207] proposed a staggered vinblastine–prednisolone protocol with an initial dosage of 2.0 mg per m^2^, increased weekly to 2.33 mg per m^2^, 2.67 mg per m^2^, and 3.0 mg per m^2^, and maintaining the last dosage at 2-week intervals. Similarly, Serra Varela and others [209] also increased the weekly dosage from 2.3 mg per m^2^ to 2.6 mg per m^2^, but the dosage of 3.0 mg per m^2^ was given weekly. In their study, the protocol was well tolerated, with 4% of patients having grade 3 or 4 toxicity. Bailey et al. [210] described the highest tolerated dose as 3.5 mg/m^2^ every 2 weeks; however, grade 4 toxicity commonly occurred among these patients. Furthermore, Klutchkovsky et al. [211] evaluated the efficacy and safety of using vinblastine at an experimental dose of 3.0 mg per m^2^ every 7 days, as compared to the conventional dose of 2.0 mg per m^2^ every 7 days. The experimental dose protocol was well tolerated, with 5.6% of the patients manifesting grade 4 neutropenia. Comparing the two groups, the animals treated with a dose of 3.0 mg per m^2^ had a lower recurrence rate, but there was no significant difference in the mean overall survival time between the groups.

Lomustine is also widely used in cases of MCT, but it is considered as a second therapeutic in terms of adjuvant chemotherapy. The recommended dose, when used as a single agent, is 60–90 mg per m^2^ every 21 days. Hosoya et al. [212] used lomustine at a dose of 60 mg per m^2^ every 3 weeks, and prednisone at a dose of 40 mg per m^2^ once a day (SID) for 7 days, followed by 20 mg/m^2^ every other day for partially resected grade 2 MCTs. The animals received treatment for 4–6 months, with no local recurrence or regional or distant metastasis, and with 100% of the animals being disease-free at 1 year and 77% being disease-free at 2 years. Hay and Larson [213] evaluated the efficacy and toxicity of the protocol using six sessions of lomustine at a dose of 70 mg per m^2^, every 4 weeks; and prednisone at a dose of 0.5–1mg per kg SID, for 6 months, for completely excised, high-grade MCTs. The results showed that the protocol was well tolerated, providing a mean overall survival time of 904 days, with 60% of dogs alive at the end of the first year and 40% of dogs alive at the end of the second year.

Protocols alternating vinblastine, lomustine, and prednisone were also tested. Cooper et al. [214] evaluated the effect of chemotherapy with lomustine at a dose of 60 mg per m^2^ and 2.0 mg per m^2^ vinblastine in dogs with grade 2 and 3 MCTs. The authors showed that this protocol was well tolerated and adequate for disease control, as the response lasted longer than that of other agents. Rassnick et al. [198] evaluated the protocol comprised of lomustine at a dose of 70 mg per m^2^, vinblastine at a dose of 3.5 mg per m^2^, and prednisone at a dose of 1–2 mg per kg in dogs with unresectable MCT, and reported observing response rates of 65% (five with CR and six with partial remission) in non-operated animals. Lejeune et al. [185] perforMed. a retrospective study to assess the clinical response of animals with grade 2 and stage 2 MCTs to the combined protocol of vinblastine at a dose of 2.0 mg per m^2^, lomustine at a dose of 60–80 mg/m^2^, and prednisone at a dose of 40 mg per m^2^, alternated every 14 days. Patients subjected to this protocol had a satisfactory disease-free time and mean survival time: 2120 days (range: 169–2325 days) and 1359 days (range: 188–2340 days), respectively.

Chlorambucil has also been used in multidrug therapy protocols, with controversial results. Taylor et al. [197] treated 21 dogs with unresectable grade 2 and 3 MCTs with a combination of prednisolone 40 mg per m^2^ SID for 14 days and 20 mg per m^2^ every other day, followed by chlorambucil at a dose of 5.0 mg per m^2^ SID. In cases where CR was achieved, treatment was stopped after 6 months; otherwise, treatment was continued. Three dogs achieved CR; nine remained in stable disease, five in partial remission, and four in disease progression (overall response rate 38%). According to the authors, this protocol yielded a worse response than other protocols. However, Horta et al. [200] observed that patients with high-risk MCT (grade 3 or grade 2 with metastasis) treated with the lomustine protocol at a dose of 60–90 mg per m^2^ every 21 days, for a total of 3–4 sessions; followed by chlorambucil at a dose of 4–6 mg per m^2^, every 48 h, for 8 weeks, reached a higher disease-free interval (686 days) than those treated only with lomustine at the maximum dose (107 days).

In addition to being part of conventional chemotherapy protocols for canine MCTs, corticosteroids are also frequently used as neoadjuvant therapy, aiming at reducing peritumoral edema and debulking the tumor. Teng et al. [215] evaluated prednisone as the only treatment for MCTs, and observed a significant reduction in tumor size, albeit with gradual resistance, as the response rate was short (81.5% of patients had a maximal response by 3 weeks). Stanclift and Gilson [196] reported an average tumor volume reduction of 80.6%, with no significant difference in the percentage reduction between the groups treated at a dose of 1.0 mg per kg and 2.2 mg per kg. Linde et al. [216] studied the effects of the use of prednisone at a dose of 1.0 mg per kg SID in canines with MCTs, in a period of 7–14 days before the biopsy. The results of the study revealed that the use of neoadjuvant prednisone in low to intermediate grade MCTs did not exert significant effects on tumor grading, mitotic count, or cell atypia. However, more studies are needed to validate these findings, and to evaluate the effects of cytoreductive therapy in reducing surgical margins.

Horta et al. [217] correlated prognostic factors with response to neoadjuvant glucocorticoid treatment in MCTs, and noted that a partial response was achieved in 63.3% of cases, while 36.7% did not respond. In their study, the response to treatment with glucocorticoids was positively correlated with the presence of already established favorable prognostic factors (patients at an early stage of the disease, with low histological grade, KIT pattern 1, and Ki-67 score < 23).

To date, few studies have been published regarding mechanisms of multiple drug resistance (MDR) related to canine MCT. Despite the use of different methods to detect genes and key proteins involved in MDR of canine MCT (MDR1, P-glycoprotein, glutathione-S-transferase pi, protein associated with multi-resistance 1, p53, etc.), no study has confirMed. the exact mechanism involved in chemotherapy resistance. Apparently, there is no histological association with the expression of MDR mechanisms that could explain why undifferentiated tumors respond poorly to chemotherapy [137,218,219,220]. It remains controversial whether the use of glucocorticoids as the only treatment for canine MCT induces MDR.

The primary outcomes of metronomic chemotherapy protocols have been studied to date [221,222]. Tripp et al. [223] evaluated a metronomic protocol using lomustine at a dose of 2.84 mg per m^2^ in different canine tumors, including MCT, and observed good tolerability in terms of adverse effects. Leach et al. [224] evaluated the metronomic protocol using chlorambucil at a dose of 4 mg per m^2^ per day for 35 weeks, in different tumor types, including four cases of canine MCT, and observed CR in one case, stable disease in two cases, and progressive disease in one case. Current knowledge about metronomic therapies indicates that their use, particularly in combination with other cytotoxic therapies, can increase response rates and optimize the treatment of cancer patients. Nevertheless, more studies are needed to validate their real effectiveness [225].

The main adjuvant and cytoreductive chemotherapy protocols (neoadjuvant and/or inoperable disease) are reported in Table 5 and Table 6, respectively.

#### 4.2.2. Tyrosine Kinase Inhibitors

The previously mentioned family of tyrosine kinase receptors are involved in the mechanisms of proliferation, differentiation, migration, angiogenesis, and cell activation [15]. Thus, studies suggest that these receptors play a fundamental role in the neoplastic transformation process [229]. The main strategy developed in veterinary medicine to inhibit these receptors was the use of the so-called “small-molecule tyrosine kinase inhibitors” (TKIs). These act by blocking the binding of ATP to the receptor by a competitive inhibition mechanism (reversible or not) preventing phosphorylation and cell proliferation and angiogenesis signaling [230]. 

The TKIs currently available in veterinary medicine are toceranib (Palladia®) and masitinib (Kinavet®/Masivet®), licensed for the treatment of unresectable or relapsed MCTs of grades II and III. There are also studies of imatinib (Gleevec®), licensed for use in humans, with a similar mechanism of action. These medications are also indicated in other circumstances, such as resistance to multiple drugs, presence of metastases, first-line adjuvant therapy for high-risk tumors, unresectable tumors, and as salvage therapy for recurrent tumors [38,229,231,232,233].

The first studies indicated that the presence of the mutations in *KIT* and the aberrant localization of the KIT protein (patterns 2 and 3) in MCTs were predictive factors of response to treatment with TKIs. London et al. [234] evaluated the efficacy of toceranib in several tumor types, and obtained response rates of approximately 90% in MCTs with mutations in the *KIT* and 25% in MCTs without such mutations. Later, London et al. [38] evaluated the effect of toceranib in dogs with relapsed or metastatic MCTs, of high or intermediate grade, and concluded that the presence of mutation in *KIT* and the absence of lymph node metastases were significantly associated with a greater response, objectively.

Although initial results indicate a positive correlation between the presence of mutation in *KIT* and response to treatment with TKIs, recent studies have indicated that the response to treatment is not associated with mutational status. In order to assess the influence of the presence of mutation in *KIT* and KIT location on the therapeutic decision, Weishaar et al. [235] compared the response of dogs with MCTs (resectable and unresectable, at various stages) to treatment with the protocol using vinblastine (2.5 mg per m^2^, four sessions every 7 days, and four sessions every 14 days) and toceranib (2.75 mg per kg, every 48 h). Mutation status in *KIT* and KIT location were not predictive of response to treatment with toceranib, and, therefore, were not considered sufficient for the therapeutic decision between toceranib and vinblastine use in dogs with macroscopic MCTs.

Therefore, it can be concluded that the presence of mutation in *KIT* is not predictive of treatment response, and that dogs with MCT that do not have *KIT* mutations can also benefit from TKIs. This is due to dysregulation in other cell pathways, particularly the platelet-derived growth factor (PDGFR) receptor, or even the anti-angiogenic effect achieved by the inhibition of the vascular endothelial growth factor receptor (VEGFR), and the possible re-establishment of antitumor immunity from the depletion of regulatory T lymphocytes and increased serum concentration of interferon-γ [38,231,232,233,236]. Other causes may be involved in the tumorigenesis of MCTs, such as the overexpression of receptors and increased production of SCF. It is also important to mention that the blockade of KIT and PDGFR occurs through the mechanisms of action of both toceranib and masitinib; however, blockade of VEGFR occurs only through the use of toceranib [36,237]. 

Other TKIs have also been studied. Horta et al. [238] evaluated the response of high-risk MCTs treated with TKIs (toceranib and masitinib) and correlated the response to treatment with different prognostic factors. In the population analyzed, the presence of mutation in *KIT*, KIT location, and Ki-67 value were not prognostic or predictors of response to TKIs. In their study, the only prognostic factor considered reliable was the initial response to treatment. Their data corroborated the study by Smrkovski et al. [239], in which the initial response to treatment was the most significant prognostic factor for dog survival. Similarly, Hahn et al. [233] evaluated the therapeutic potential of masitinib in dogs with MCT, noting that there was an increase in the time to disease progression, regardless of the presence or absence of mutations in *KIT*.

The ideal doses of TKIs have also been investigated. The commercial indication for toceranib dosage is 3.25 mg per kg on Mondays, Wednesdays, and Fridays. There is clinical evidence of good efficacy and greater safety at lower doses of toceranib. London et al. [234] perforMed. the first study on the efficacy and safety of toceranib in dogs with different tumor types using staggered doses, ranging from 1.25 to 3.75 mg per kg, every 48 h. In this study, dogs treated with a dose of 2.5 mg per kg every 48 h had a similar response and fewer adverse effects than dogs treated with a dose of 3.25 mg per kg. London et al. [38] used toceranib at a dose of 3.25 mg per kg every 48 h, in dogs with recurrent and metastatic MCTs, obtaining an objective response of 42.8%, and with 34.5% of patients developing grade 3 to 4 adverse effects. A dosage of 2.5 to 2.75 mg per kg, every 48 h, can be advantageous due to its greater safety, better adherence by owners, and a lower probability of breaks during treatment [240]. 

Smrkovski et al. [239] evaluated the efficacy of masitinib in the treatment of grade 2 and III MCTs, at a dose of 12.5 mg per kg, as rescue therapy or first-line treatment, and obtained a mean survival time of 630 days. In contrast, in the study by Grant et al. [241], dogs with macroscopic MCTs treated with masitinib at a dose of 12.5 mg per kg had a mean survival time of only 159 days, considering that, in most cases, masitinib was instituted as rescue therapy.

Recently, the efficacy and safety of the association of TKIs with other conventional chemotherapeutics have been evaluated, in order to maximize their antitumor effects. To determine the maximum tolerated dose, the study by Robat et al. [242] reported that the use of vinblastine (1.6 mg per m^2^ every 14 days) and toceranib (3.75 mg per m^2^ every 48 h) was well tolerated, with significant activity against MCT (71.4% response objective). Olsen et al. [229] evaluated the efficacy of the protocol of vinblastine (1.6 mg per m^2^, every 14 days, in 8 sessions), prednisolone (1.0 mg/kg SID), and toceranib (2.5 mg per m^2^, every 48 h, for 16 weeks) as neoadjuvant therapy for large MCTs and as adjuvant to surgery for high-grade MCTs, resulting in an 88% response in the neoadjuvant therapy group and a mean survival time of 893 days in the adjuvant therapy group.

The therapeutic combination of TKIs with lomustine was also evaluated. Pan et al. [243] carried out a study in dogs with different tumors, combining the use of toceranib (2.75 mg per kg, every 48 h) and lomustine, with a defined maximum tolerated dose of 50 mg per m^2^, every 3 weeks. Similarly, Burton et al. [195] reported that the maximum tolerated dose of lomustine, when combined with toceranib (2.75 mg per kg, days 1, 3, and 5 of a 21-day cycle) was also 50 mg per m^2^, with an objective response rate of 46% in unresectable MCTs. In both studies, the protocol was well tolerated, the most common adverse effect being neutropenia, with an increase in alanine transaminase enzyme levels in more than 50% of patients. Additionally, Bavcar et al. [244] observed that the protocol combining toceranib (2.7 mg per kg, every 48 h), lomustine (60 mg per m^2^, every 21 days), and prednisolone (1.0 mg per kg, every 48 h, alternating with the toceranib) had a 50% satisfactory objective response rate, but resulted in significant toxicity, leading to the need for some protocol change in all study animals, and with 30% deaths/euthanasia due to therapy-related toxicity. In that study, neutropenia was also the most frequent adverse effect. The association of TKIs with conventional chemotherapy has already been proven to be effective and safe, if tolerable doses are respected, but in some cases, it can cause serious adverse effects. 

Similar to chemotherapeutic agents, TKIs also have adverse effects [38]. These effects result from chronic inhibition of the receptor tyrosine kinase in normal cells, which depend on these kinases for survival and proliferation under normal conditions. The main adverse effects are gastrointestinal problems and neutropenia, but dermatological signs, hepatotoxicity, nephrotoxicity, hypoalbuminemia, and hypertension have also been reported [230]. In the face of adverse effects, the dose of toceranib can be reduced by 0.5 mg per kg (up to a minimum dose of 2.2 mg per kg) and treatment can be interrupted for up to 2 weeks, according to the manufacturer’ recommendations. Importantly, the response to TKIs may be slower and, therefore, maintenance of the medication for at least 3 weeks is suggested. However, in cases of progressive disease, the duration of treatment should be reassessed. 

The authors of this consensus guideline recommend treatment with TKIs, particularly for patients with unresectable or metastatic cutaneous MCTs or as adjuvant therapy (alone or associated with chemotherapy). However, given the absence of predictive factors for the response to TKIs and chemotherapy, it is indicated that the choice of treatment should be based on each patient’s individual data, considering the clinical criteria (size, ulceration, growth rate), histopathological data (grading by Kiupel and Patnaik, mitotic count, and lymph node status), and as necessary, immunohistochemical data (KIT pattern and Ki-67 count).

### 4.3. Support and Palliative Treatment

As previously mentioned, the clinical signs most commonly associated with MCTs are directly related to mast cell degranulation, mainly leading to gastrointestinal ulcerations, delayed wound healing, hypotensive shock, and coagulopathies. Therapeutic management of these complications through supportive therapies is employed to prevent and minimize these deleterious systemic effects, improving the quality of life of patients [15,67]. With a view to prevention and treatment of systemic signs caused by hyperhistaminemia, the use of histamine receptor inhibitors (H1 and H2) is indicated. To prevent and treat gastrointestinal ulcerations, the use of H2 inhibitors is recommended (famotidine, at a dose of 0.5–1 mg per kg, BID; cimetidine, at a dose of 4–5.5 mg per kg TID; or ranitidine, at a dose of 2 mg per kg, BID). Occasionally, dog’s refractory to antihistamine therapy may benefit from the use of proton pump inhibitors (omeprazole, at a dose of 0.5–1 mg per kg, BID–SID), as they also inhibit gastric acid production, but via a proton bomb effect. In cases where there is evidence of active gastrointestinal ulceration, sucralfate can be added to the protocol, at a dose of 0.5–1 g BID [14,132]. H1 inhibitors (diphenhydramine, at a dose of 2–4 mg/kg BID) are indicated to minimize the adverse effects of histamine release on the peripheral vasculature and wound healing, as well as for the treatment of erythema, edema, and pain locations [67]. In cases with a higher degree of inflammation, local pain management can also be achieved with the use of analgesics and systemic anti-inflammatory drugs.

It is important to emphasize that the release of histamine by tumor manipulation during surgery can lead to unwanted cardiovascular effects, such as a decrease in vascular resistance, systemic blood pressure, and the development of arrhythmias, as well as hypersensitivity reactions and, more rarely, anaphylactic shock. Therefore, the use of H1 inhibitors (diphenhydramine 1–2 mg per kg, SC) should be instituted for dogs with MCTs at 30–60 min before the procedure [66,132,245,246]. Furthermore, some anesthetic drugs, such as some opioids (morphine and meperidine), have the potential to maximize histamine release, and should be avoided in patients with MCTs [247].

In addition to histamine, other vasoactive substances may also be involved. Heparin release by neoplastic mast cells mainly contributes to the development of local coagulopathies. Heparin released during surgical manipulation of the tumor can also lead to considerable bleeding. Protamine sulfate, a heparin antagonist, is indicated for use in cases of prolonged bleeding [248].

Therefore, this consensus indicates the institution of supportive therapeutic management in patients with MCTs who:

1. Manifest systemic and/or local clinical signs;
2. Are larger;
3. Will undergo considerable surgical manipulation or tumor section (cytoreductive surgery);
4. With a greater probability of local degranulation (chemotherapy or radiotherapy in macroscopic disease); and/or
5. Patients with metastatic disease [15,67,132,245].

## 5. Future Perspectives

New studies are underway to test new therapies that can improve the treatment of canine MCT. Researchers have evaluated the efficacy of therapies by the intramuscular and intratumoral routes, using an electrogene with plasmid encoding interleukin-12 (IL-12), resulting in CR in 2/3 canines with MCT, and volume reduction in about 83% of the tumor [249].

Advances in epigenetics have shown the existence of histone hypoacetylation in cancer. Thus, experimental treatments with a histone deacetylation inhibitor, AR-42, induced growth reduction and apoptosis in vitro in a strain of canine MCT, which may be a promising therapy for this tumor [250]. A Brazilian study evaluated Trichostatin A (TSA), a histone deacetylase inhibitor that has an antiproliferative effect and induces apoptosis in cancer cells. The action of the drug in vitro on MCT grade III cells had deleterious effects on the growth and proliferation of tumor cells, suggesting a good chemotherapeutic potential [251]. Furthermore, the JAK2/STAT5 signaling pathway has been the target of studies in different neoplastic types, with evidence of its activity in cases of canine MCT, representing a possible target for the development of new therapies [252,253].

## Figures and Tables

**Table 1:** Clinical Staging System for Canine Cutaneous MCT proposed by the WHO.

Stage	Description
**0**	One tumor incompletely excised from the dermis, identified histologically, without regional lymph node involvement. a. Without systemic signs. b. With systemic signs.
**I**	One tumor confined to the dermis, without regional lymph node involvement. a. Without systemic signs. b. With systemic signs.
**II**	One tumor confined to the dermis, with regional lymph node involvement. a. Without systemic signs. b. With systemic signs.
**III**	Multiple dermal tumors; large, infiltrating tumors with or without regional lymph node involvement. a. Without systemic signs. b. With systemic signs.
**IV**	Any tumor with distant metastasis, including blood or bone marrow involvement.

MCT, mast cell tumor; WHO, World Health Organization.

**Table 2:** Clinical Staging System for Canine Cutaneous MCT proposed by SEVC.

Stage	Tumor	Lymph Node	Metastasis
**I**	Single nodule, <3 cm, well circumscribed.	−	−
**II**	+1 nodule, <3 cm, intralesional distance > 10 cm, well circumscribed.	−	−
**III**	1 or + nodule, >3 cm, intralesional distance < 10 cm, poorly circumscribed or ulcerated.	−	−
**IV**	Any type of lesion.	+	−
**V**	Any type of lesion.	+ or −	+

SEVC, Southern European Veterinary Conference; MCT, mast cell tumor

**Table 3:** Clinical Staging system for Canine Cutaneous Mast Cell Tumors proposed by Horta et al. [27,11], Adapted from the World Health Organization System.

Stage	Description
**I**	Single tumor, without regional lymph node involvement.
**II**	Multiple tumors (≥ 3), without regional lymph node involvement.
**III**	Single tumor, with regional lymph node involvement.
**IV**	Large and infiltrative tumors, without delimitation, or multiple tumors (≥ 3), with regional lymph node involvement.
**V**	Any tumor with distant metastasis, including bone marrow invasion and the presence of mast cells in the peripheral blood.

**Table 4:** Prognostic Factors Associated with Canine Cutaneous MCT.

Prognostic Factor	Positive	Negative	Reference
Histopathological Grading	Grade 1–low Grade	Grade 3–high Grade	Patnaik et al. [47,48]; Kiupel et al. [80]
History of Tumor Recurrence	First clinical presentation	Recurrent tumor	Horta et al. [11]
Tumor Size	Higher diameter < 3 cm	Higher diameter > 3 cm	Hahn et al. [134]
Metastasis (regional and/or distance)	Absent	Present	Hume et al. [51]; Book et al. [121]; Warland et al. [7,122]
Surgical Margins	Free margins	Contaminated margins	Ozaki et al. [133]
Mitotic Count	<5 or <7	>5 or >7	Romansik et al. [92]; Elston et al. [93]
KIT Pattern	KIT 1	KIT 2 and 3	Kiupel et al. [19]
*c-KIT* Mutation*c-KIT* Mutation	Absent	Present	Webster et al. [32,35,36]
Ki67 Index	<23	>23	Webster et al. [32,139]

**Table 5:** Chemotherapy Protocols Evaluated in Canine Mast Cell Tumors and Mean Overall Survival.

Chemotherapeutic Protocol	*n*	Histopathological Grading	Overall Survival Median
Prednisolone and Vinblastine [168,169]	61 (a, b)	a. Grade 2/3 b. Grade 3	a. 1374 days b. 800 days
Prednisone and Vinblastine [179,180]	41	Grade III and Grade 2/High grade	904 days
Prednisolone, Vinblastine and Lomustine [149,150]	21	Grade 2	1359 days
Lomustine and Vinblastine [180,181]	20	Grade 2/3	336 days

**Table 6:** Published Chemotherapy Protocols for Treating Advanced Mast Cell Tumors in Dogs and the Associated Remission Rates (RR) (Adapted from [162,197]).

Chemotherapeutic Protocol	*n*	RR (C + P)
Prednisone/Prednisolone [216]	60	63%
Prednisone [195,196]	49	70%
Prednisone [215,216]	16	63%
Prednisone and Vinblastine [225,226]	41	47%
Prednisolone, Vinblastine, and Cyclophosphamide [226,227]	35	63%
Prednisone, Vinblastine, and Lomustine [197,198]	35	65%
Prednisone, Vinblastine, and Lomustine [213,214]	56	57%
Lomustine [227,228]	19	40%
Chlorambucil and Prednisolone [196,197]	21	38%

## Data Availability

Data are available upon request.
